# Mechanistic Approach to Stability Studies as a Tool for the Optimization and Development of New Products Based on *L. rhamnosus* Lcr35® in Compliance with Current Regulations

**DOI:** 10.1371/journal.pone.0079041

**Published:** 2013-11-11

**Authors:** Claudia Muller, Virginie Busignies, Vincent Mazel, Christiane Forestier, Adrien Nivoliez, Pierre Tchoreloff

**Affiliations:** 1 Département Recherche et Développement, Probionov, Aurillac, France; 2 Laboratoire Matériaux et Santé EA401, Univ. Paris Sud, Châtenay-Malabry, France; 3 Laboratoire Microorganismes : Genome Environnement (LMGE) UMR CNRS 6023 Univ. d’Auvergne-Clermont 1, Clermont-Ferrand, France; Wageningen University, Netherlands

## Abstract

Probiotics are of great current interest in the pharmaceutical industry because of their multiple effects on human health. To beneficially affect the host, an adequate dosage of the probiotic bacteria in the product must be guaranteed from the time of manufacturing to expiration date. Stability test guidelines as laid down by the ICH-Q1A stipulate a minimum testing period of 12 months. The challenge for producers is to reduce this time. In this paper, a mechanistic approach using the Arrhenius model is proposed to predict stability. Applied for the first time to laboratory and industrial probiotic powders, the model was able to provide a reliable mathematical representation of the effects of temperature on bacterial death (R^2^>0.9). The destruction rate (k) was determined according to the manufacturing process, strain and storage conditions. The marketed product demonstrated a better stability (k = 0.08 months^−1^) than the laboratory sample (k = 0.80 months^−1^). With industrial batches, k obtained at 6 months of studies was comparable to that obtained at 12 months, evidence of the model’s robustness. In addition, predicted values at 12 months were greatly similar (±30%) to those obtained by real-time assessing the model’s reliability. This method could be an interesting approach to predict the probiotic stability and could reduce to 6 months the length of stability studies as against 12 (ICH guideline) or 24 months (expiration date).

## Introduction

Probiotics are defined by the World Health Organization (WHO) as viable live microorganisms, which, when administered in adequate amounts, confer a health benefit on the host [1]. Because of their large potential applications in human diseases such as gastrointestinal disorders, immunomodulation or cholesterol reduction, probiotic products are of great interest to the pharmaceutical industry [2]–[6]. The most commonly used strains for applications in both the food and pharmaceutical industries belong to the *Lactobacillus* and *Bifidobacterium* bacterial genus [4], [7].

In addition to having beneficial health effects, probiotic bacteria must possess the properties required to be used as pharmaceutical products, such as safety, genetic stability, ability to be produced on a large scale, product stability (viability), and acceptable flavor or taste [5], [7]–[9]. One of the most important criteria is the maintenance of viability during the product life cycle [5], [10], which can be affected by the culture medium, manufacturing process and residual humidity [11]–[17].

Depending on the application (food or pharmaceutical) and the target (disease), the number of viable bacteria in the probiotic can vary, and has to be previously established by the producer [3], [9], [15]. The method used for determining the stability of pharmaceutical products is defined by the ICH Guideline Q1A [18] and requires a minimum of 12 months to test products under different storage conditions (Table 1). The stability studies can be performed until the expiration date (24 or 36 months) leading to a time-consuming method. Faced with the increasing cost of laboratory technology, producers would welcome any new strategy that could offer savings.

**Table 1:** Storage conditions requested by guideline ICH Q1A for the development of new drug products.

Study	Storage condition	Minimum time period covered by data at submission
Long term*	25°C ±2°C/60% RH ±5% RH or 30°C ±2°C/65% RH ±5% RH	12 months
Intermediate**	30°C ±2°C/65% RH ±5% RH	6 months
Accelerated	40°C ±2°C/75% RH ±5% RH	6 months

* It is up to the applicant to decide whether long term stability studies are performed at 25°C ±2°C/60% RH ±5% RH or 30°C ±2°C/65% RH ±5% RH.

** If 30°C ±2°C/65% RH ±5% RH is the long-term condition, there is no intermediate condition.

Previous studies have shown the interest of a mechanistic approach in predicting the stability of active pharmaceutical ingredients (API) in formulations, especially for chemical molecules, in vitamin denaturation [19], [20]. Data predictions using an appropriate kinetic model could shorten the time required for a stability study. The Arrhenius equation has been widely applied for this purpose [19], [21], [22]. The ICH Q8 guideline outlines how time can be saved during product development with the aid of Design Space, which can be performed with a study design or with a mathematical equation [23].

Just like chemical compounds, bacteria are affected by increases in temperature. Accelerated storage conditions (40°C) lead to a greater loss of viability than long-term storage conditions or storage at room temperature [14], [19], [24], [25]. The stability and the kinetic constant (k) depend on temperature but also on the formulation, manufacturing process and intrinsic resistance of the bacterial strain [8], [14], [26]. Each formulation should have its own equation. A new constant k obtained in real time can be compared with a reference kinetic to anticipate possible problems in the manufacturing of new industrial batches or during laboratory trials.

This paper analyses the stability of the probiotic strain *Lactobacillus rhamnosus* Lcr35® in manufactured powders stored at different temperatures. Isolated from human intestinal microbiota, this probiotic strain is used for intestinal and gynecological applications to restore flora after a disruption such as antibiotic treatment or stress [27], [28]. Its biological and physical properties including stability during manufacture and storage have been described elsewhere [27], [29]–[31]. We assessed the ability of the Arrhenius model to determine the viability of *L. rhamnosus* Lcr35® at different storage temperatures using laboratory and industrial powders. We obtained a specific Arrhenius equation to predict stability irrespective of storage length and temperature. The repeatability and the robustness of this approach were verified by comparing predicted and observed stability values from industrial batches.

## Materials and Methods

### 1. Microorganisms and Growth Production

For the laboratory assay, the probiotic bacteria *L. rhamnosus* Lcr35®, derived from reference cryotubes (Probionov, Aurillac, France) containing the native strain. *L. rhamnosus* Lcr35®, was grown in Man, Rogosa, Sharpe (MRS) broth (AES, Bruz, France) for 48 h at 37°C (three samples). Reconstituted milk was added (110 g/l) as cryoprotectant before lyophilization. This medium composed of MRS and reconstituted milk, will hereafter be designated “critical medium”. The resulting bacterial suspensions were then lyophilized and the obtained cakes were ground in a Magimix® blender. The powders obtained from the laboratory assay were packaged in a glass bottle with a hermetic plug.

An industrial product containing *L. rhamnosus* Lcr35®, manufactured and provided by Probionov (Aurillac, France), was also included in the study. Its commercial name is Bacilor® and it contains the active pharmaceutical ingredient (API) *Lcr restituo®*. Eleven batches of Bacilor® (*Lcr restituo®*) capsules manufactured between 2006 and 2011 and one manufactured in 2012 were taken to make comparisons between predicted and observed stability values.

### 2. Storage Conditions and Stability Testing (viability of *Lactobacillus*)

Powders obtained with the critical medium and the 12 industrial batches of Bacilor® (*Lcr restituo®* capsules) were stored and checked as recommended by the ICH Guideline Q1A (Table 2). At each checkpoint, the number of viable *Lactobacillus* was determined by a plate count method as follows. The powders were re-suspended in peptone water (Pastone 1 g/l, Biorad, Marnes-la-coquette, France) and a serial 10-fold dilution was performed. Each dilution was plated on MRS agar (Biomérieux, Marcy l’Etoile, France) plates and incubated at 37°C for 72 h. The results were then expressed as colony forming units (CFU.g^−1^).

**Table 2:** Stability testing for the products according to the ICH Guideline Q1A.

	20°C, 25°C, 30°C	40°C
Laboratory assay	0, 3, 6, 9, 12	/
Eleven batches	0, 3, 6, 9, 12, 18, 24	/
Recent batch	0, 1, 3, 6	0, 1, 2, 3, 4, 5, 6

Storage conditions and check points (months) realized on powders obtained with the laboratory assay and the industrial batches (Bacilor®).

### 3. Linear-Arrhenius Model

A standard two-step method was used to obtain the Arrhenius model and to assess the influence of temperature on the stability of *Lactobacillus rhamnosus* Lcr35®.

Predictive microbiology describes the exponential loss of bacterial viability over time by the following equation (first-order low):
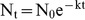
(1)


t: time.

k: destruction rate.

N_0_: number of viable microorganisms at t = 0.

N_t_: number of viable microorganisms at t = t.

By plotting ln (N_t_/N_0_) over time t, k can be determined for each temperature. The effect of temperature on k can be represented by the Arrhenius equation:
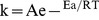
(2)


k: destruction rate (time^−1^).

A: frequency factor (time^−1^).

R: gas constant (8,314 J.mol^−1^.K^−1^)

T: temperature (Kelvin).

Ea: activation energy (J.mol^−1^).

By plotting ln(k) versus 1/T, a straight line is obtained, which leads to the parameters of equation (2). By extrapolation, k is accessible for any temperature. Using equation (1), the number of viable microorganisms can be predicted at any time and for any storage condition.

Finally, k is also related to the decimal reduction time D_1_, the time for which the initial bacterial population is reduced by 90% [19]. D_1_ is defined as:
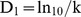
(3)


By extension, it is possible to define the time for which the initial bacterial population is reduced by 99 or 99.9% (D_2_ and D_3_).

## Results and Discussion

### 1. Model Validation on Laboratory Powders

The powders obtained from the laboratory assay were used to validate the use of the Arrhenius model in assessing bacterial stability over a 12-month period. The critical medium does not provide high bacterial stability during product process and storage (data not shown) and was therefore chosen as a model of damaged stability conditions.

**Figure 1:**
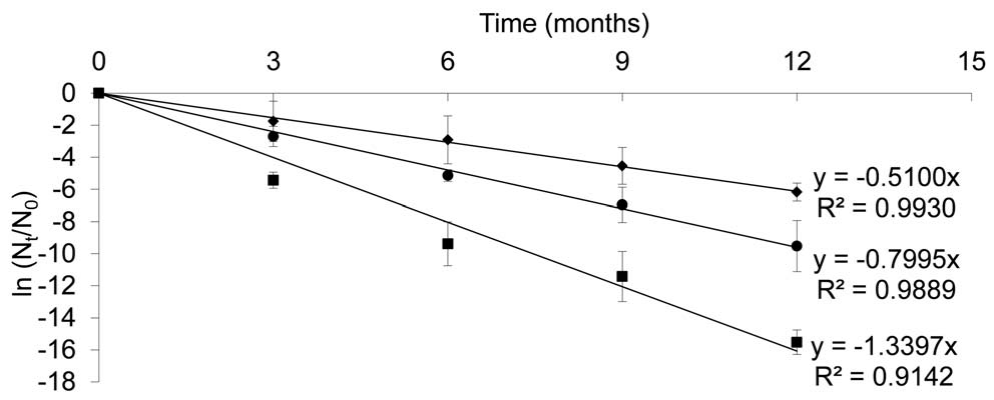
Effect of storage conditions on bacterial stability within the laboratory powder. Plot of ln(N_t_/N_0_) for powders obtained with the laboratory assay against time and temperature 20°C (♦), 25°C (•) and 30°C (▪) (Mean of three values), according to the current regulation (ICH Guideline Q1A).

The destruction rate k was determined at three storage temperatures (20, 25 and 30°C) using equation (1) (Fig. 1). When the temperature increased so did the k values (k_20°C_<k_25°C_<k_30°C_), which demonstrates, in agreement with Davey’s study [19], that, due to the sensitivity of the microorganisms to heat, viability was lost (Table 3). The values of decimal reduction times D1, D2 and D3, unlike the destruction rate, decreased as the temperature increased. At 20°C, it took 4.5 months to reduce the initial bacterial population by 90% whereas at 30°C only 1.7 months was required. Thus, the loss of bacterial viability was dependent on temperature (k values) and the length of storage (D values).

**Table 3:** Kinetic parameters of the laboratory powders.

Temperature (°C)	k (months^−1^)	Decimal reduction time (months)		
		D_1_	D_2_	D_3_
20	0.51	4.5	9.0	14
25	0.80	2.9	5.8	8.6
30	1.34	1.7	3.4	5.2

Destruction rates (k) and decimal reductions (D) obtained for the powder of the laboratory assay (values obtained with the mean of stability studies performed on three assays).

**Figure 2:**
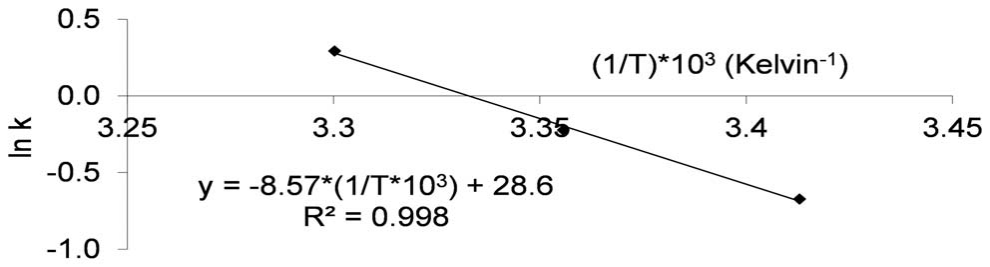
Arrhenius representation for the laboratory powder (Lcr35® bacteria). The observed k values were previously obtained with ln(N_t_/N_0_) against time for the laboratory powder (Fig. 1). These k values (y axis) were represented against 1/T (Kelvin^−1^) (x axis) to obtain the Arrhenius equation of this assay (Equation 4).

The obtained Arrhenius model was plotted (Fig. 2). k was related to temperature by the following Arrhenius equation:
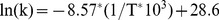
(4)With this mathematical relation, k could be extrapolated to a range of temperatures to obtain its variation under different storage conditions. Activation energy (Ea) extracted from this equation was 71 kJ.mol^−1^. The Arrhenius equation obtained from experimental data was applied to the ln (N_t_/N_0_) data with a high correlation (R^2^ = 0.998), which suggests that the model is suitable to determine the loss of bacterial viability in different storage conditions.

### 2. Application on Industrial Batches: Bacilor® (Lcr restituo®) Capsules

#### 2.1 Determination of the arrhenius equation for the 11 batches (2006–2011)

A mean of the stability values obtained for the 11 batches of Bacilor® (*Lcr restituo®*) capsules was calculated at each checkpoint. The Arrhenius model was then applied to these mean values. The guideline stipulates a minimum study time of 12 months, but stability can be tested as long as needed (i.e. until expiration date), sometimes up to 36 months. The industrial batches tested in this study were monitored over 24 months at three temperatures (20, 25, 30°C).

**Figure 3:**
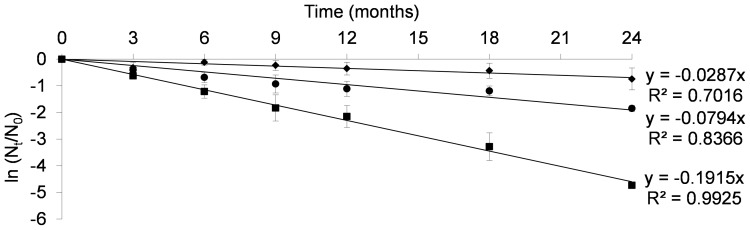
Effect of storage conditions on bacterial stability within Bacilor® (*Lcr restituo®* capsules). Plot of ln(N_t_/N_0_) for the batches of Bacilor® (*Lcr restituo®* capsules) against time and temperature 20°C (♦), 25°C (•) and 30°C (▪) (Mean of eleven values), according to the current regulation (ICH Guideline Q1A).

As shown in Figure 3, k increased with the temperature, but the values were about 10 times lower than those obtained previously with the laboratory powders (at 25°C, k_(Bacilor)_ = 0.08/k_(critical media)_ = 0.80 months^−1^) (Table 4). The powders obtained with the critical medium led to low D values and a high destruction rate (k) whatever the temperature (D_1(20°C) = _4.5 months, k_20°C = _0.51 months^−1^). The industrial batches were less affected by temperature (D_1(20°C)_ = 80.2 months, k_20°C = _0.03 months^−1^), demonstrating their better stability over time. The failure of the critical medium to promote bacterial stability during manufacturing and storage was thus confirmed. In agreement with Oliveira et al. [15], our results illustrate the real influence of formulation on maintaining bacterial viability during these two phases. The use of lyophilization to dry the fermented culture media is one of the most useful processes to maintain optimal bacterial viability during product process and storage. However, as shown by the different results obtained with laboratory samples and industrial powders, the culture growth medium also has an important role in maintaining stability and must be carefully formulated during product development. Depending on the nature of the cryoprotectants and the culture components, the stability of any given bacterial strain can differ according to the stress encountered during the product’s life cycle [13], [14], [16], [17], [32]. The impact of any modification (excipient, formulation or bacterial strain) on product stability could therefore be easily detected by determining the k value and comparing it with a reference kinetic.

**Table 4:** Kinetic parameters of Bacilor® (*Lcr restituo®* capsules).

Temperature (°C)	k (months^−1^)	Decimal reduction time (months)		
		D_1_	D_2_	D_3_
20	0.03	80.2	161.0	241.0
25	0.08	29.0	58.0	87.0
30	0.19	12.0	24.1	36.1

Destruction rates (k) and decimal reductions (D) obtained with the commercial product powders (values obtained with the mean of stability studies performed on eleven batches of Bacilor® (*Lcr restituo®* capsules)).

**Figure 4:**
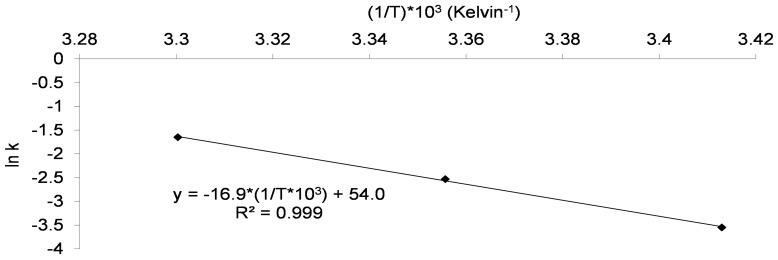
Arrhenius representation for Bacilor® (*Lcr restituo®* capsules). The observed k values were previously obtained with ln(N_t_/N_0_) against time for Bacilor® (*Lcr restituo®* capsules) (Fig. 3). These k values (y axis) were represented against 1/T (Kelvin^−1^) (x axis) to obtain the Arrhenius equation of this assay (Equation 5).

The Arrhenius model was also applied to the results on the stability of the 11 batches (Fig. 4), and gave the following equation:
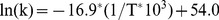
(5)


The equation yielded an activation energy (Ea) of 140 kJ.mol^−1^ higher than that obtained with the laboratory powders (Ea = 71 kJ.mol^−1^), evidence that bacterial stability and product formulation are closely interdependent.

The straight line of the equation showed a high correlation (R^2^ = 0.999) and evidenced the relation between the loss of bacterial viability (k) and the storage temperature of industrial batches. From equation (5), k was calculated for accelerated storage conditions (40°C) and had a value of 1.15 months^−1^.

#### 2.2 Application on a recent industrial batch (2012)

**Figure 5:**
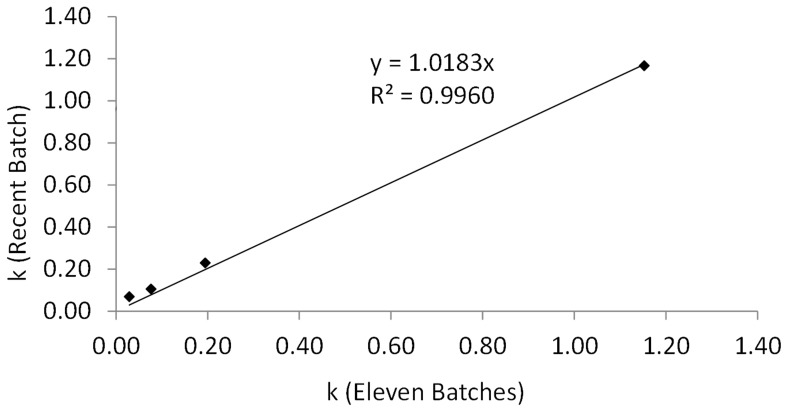
Comparison of the obtained and predicted k values. Observed k values obtained with stability studies of the recent batch during six months, against the k values obtained with the Arrhenius equation of the eleven batches of Bacilor® (*Lcr restituo®* capsules) (the k value at 40°C for the eleven batches was not available and was predicted with the Arrhenius equation).

To assess the accuracy of this model, k values obtained with stability studies of the 11 batches were compared with those obtained with the recent batch. Stability values were recorded at four temperatures (20, 25, 30 and 40°C) during a 6-month follow-up, which gave k_20°C_<k_25°C_<k_30°C_<k_40°C,_ as previously shown. Concerning the loss of viability, k observed at 40°C for the recent batch (k_40°C = _1.17 months^−1^) was closely similar to that predicted for the old batches of Bacilor® (k_40°C = _1.15 months^−1^). Comparison of these values yielded a guiding coefficient equal to 1.0183 (R^2^ = 0.996) (Fig. 5).

**Figure 6:**
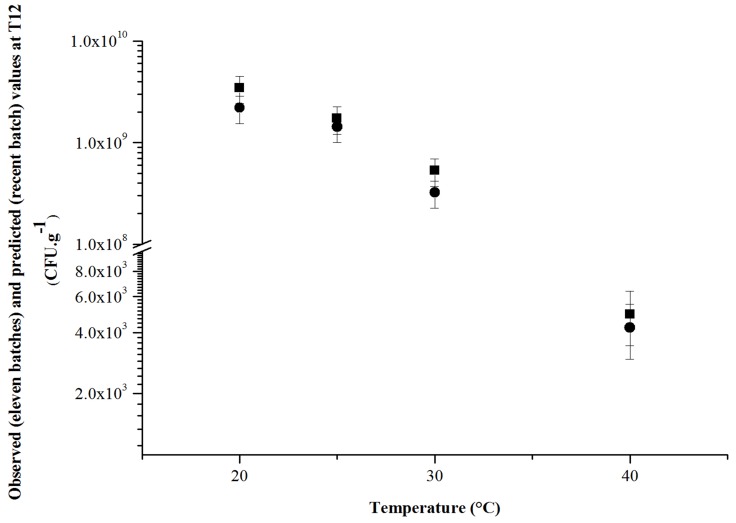
Comparison of observed and predicted stability values. Comparison between observed values (mean of 11 values) at 12 months on the eleven batches of Bacilor® (*Lcr restituo®* capsules) (▪) and predicted values at 12 months for the recent one (•) against temperature.

To assess this correlation, the number of viable lactobacilli in the recent batch was determined at 12 months (Equation 1) and compared with those of the 11 other batches (Fig. 6). A standard deviation of 30%, due to biological variability, sampling and dilution, is commonly accepted and was added to each value [33], [34]. In spite of this constraint, it was clearly shown that whatever the temperature, the observed and predicted numbers of viable lactobacilli were closely similar after 12 months. A stability study performed during the 6 first months of storage gave very similar Arrhenius parameters to those in a stability study performed over 12 months in accordance with the ICH guideline Q1A. Thus, 6-month stability studies performed at three temperatures (Arrhenius representation) should be sufficient to predict the behavior of the product as successfully as a 12-month study (ICH Q1A) or even longer ones (up to expiration date).

As previously shown for chemical APIs [19], [20], stability studies of probiotic products can be improved by data predictions using the Arrhenius equation [35]. It would therefore be possible to predict the behavior of the product under storage conditions before the end of the follow-up period. In the intermediate and long-term, problems of product quality or stability (i.e. problems encountered during the development process) could be detected and resolved earlier as the QbD approach (ICH Q8).

## Conclusion

In the pharmaceutical industry, regulations are more restrictive than in the food industry, owing, in great part, to the ICH Q1A, which imposes check-ups over 12 months for each new product. The present work is the proof that the follow-up period needed to perform stability studies can be reduced and the results anticipated by using the Arrhenius model.

The stability measurements obtained in our study were similar for commercial batches of *Lactobacillus rhamnosus* Lcr35® produced at different times. This demonstrates the robustness of the model, which could be widely applied to probiotic products to predict the influence of different temperatures at any time.

Probiotics must be viable at sufficient dosage levels at the time of consumption and until their expiration date to have a health effect. As for sterilization, the manufacturers could determine the time at which viability meets the regulatory standards. The destruction rate obtained in real time for a new product could be compared with a reference kinetic to identify any change in formulation, bacterial strain or manufacturing process. Applied to probiotic products, this approach could be of great value in their development and market approval.

To conclude, reducing the time dedicated to research and development is one of the most important challenges for the pharmaceutical industry (ICH Q8). This aim could be achieved in compliance with ICH Q1A guidelines by using the Arrhenius model in stability studies.
